# Supplementary material for: Precise measurement of molecular phenotypes with barcode-based CRISPRi systems
Source: bioRxiv. 2024 Jun 22:2024.06.21.600132. Preprint. [Version 1] doi: 10.1101/2024.06.21.600132 (PMC11213135; doi:10.1101/2024.06.21.600132)
Supplement: Supplement 1 [file NIHPP2024.06.21.600132v1-supplement-1.pdf]

bioRxiv preprint doi: https://doi.org/10.1101/2024.06.21.600132; this version posted June 22, 2024. The copyright holder for this preprint (which was not certified by peer review) is the author/funder, who has granted bioRxiv a license to display the preprint in perpetuity. It is made available under aCC-BY 4.0 International license.

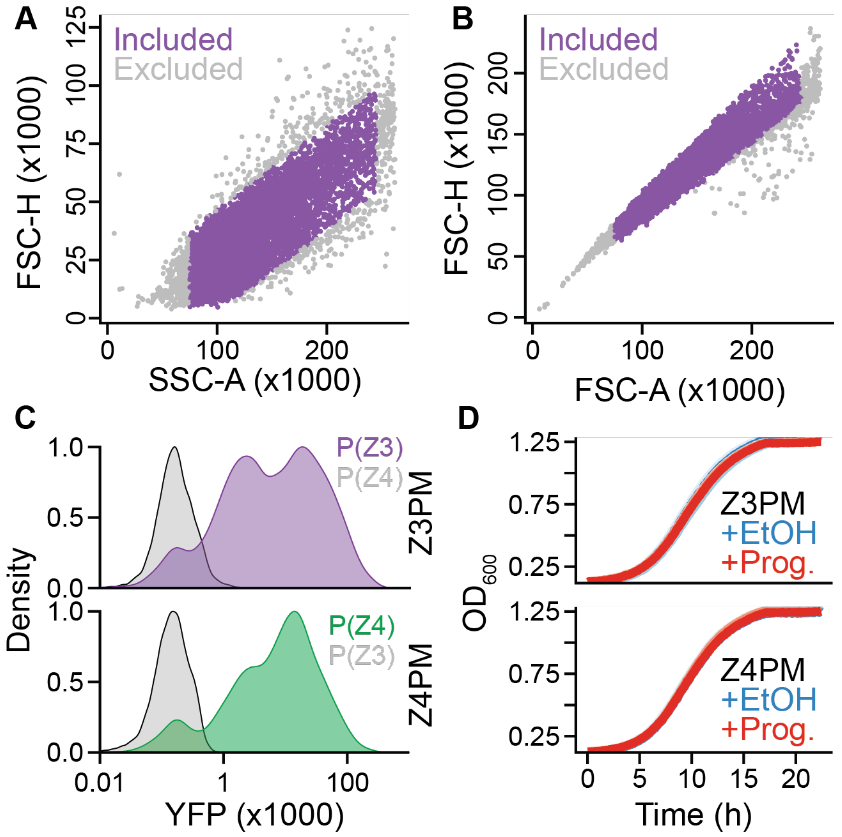

Figure S1: Evaluation of Z3PM and Z4PM for reporter expression. (A-B) Representative gating criteria for forward and side scatter in all flow cytometry analysis. **(C)** Representative raw flow cytometry histograms of Z3PM or Z4PM driving YFP expression from P(Z3) or P(Z4) at 200 nM progesterone. **(D)** Raw growth curves from yeast containing Z3PM or Z4PM and expressing YFP from the cognate promoter, with 200 nM progesterone or matched volume of ethanol in synthetic complete media lacking uracil (n=3).

bioRxiv preprint doi: https://doi.org/10.1101/2024.06.21.600132; this version posted June 22, 2024. The copyright holder for this preprint (which was not certified by peer review) is the author/funder, who has granted bioRxiv a license to display the preprint in perpetuity. It is made available under aCC-BY 4.0 International license.

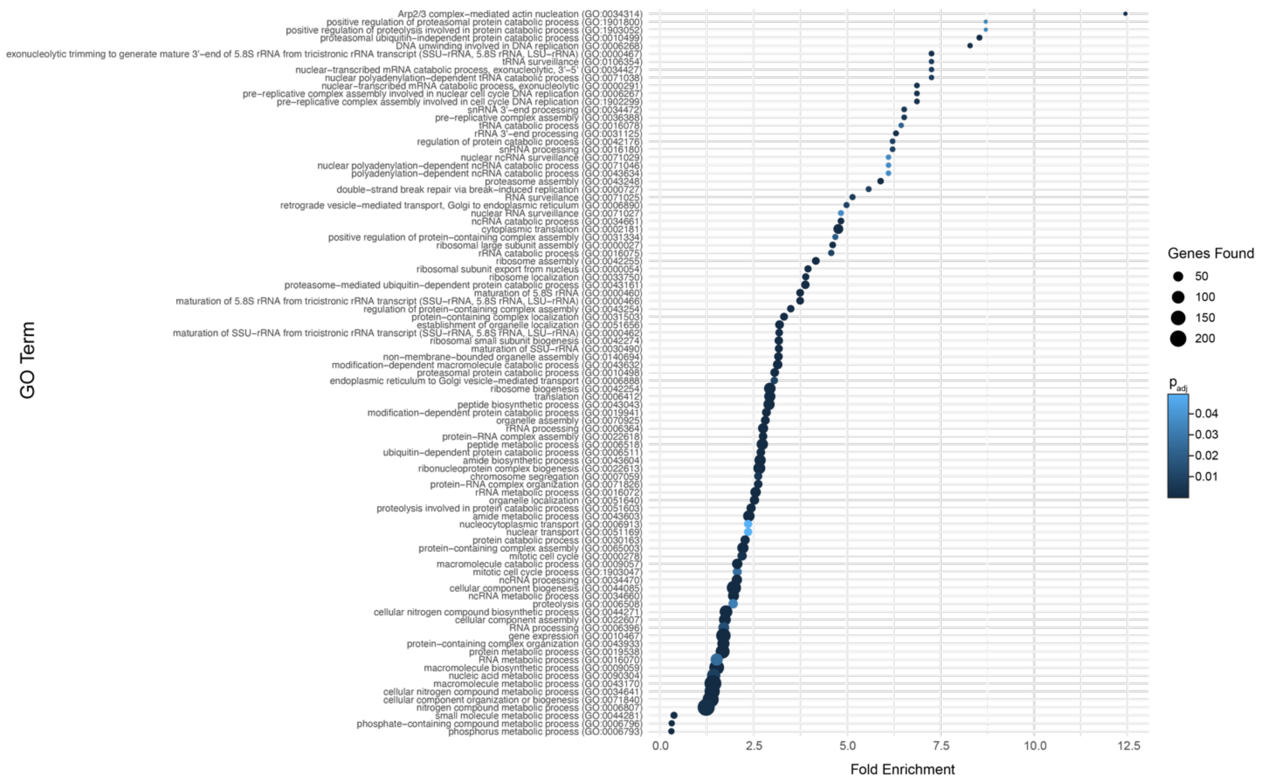

Figure S2: Analysis of significant processes in DNA-normalized CiBER-seq. Gene ontology terms for guides that significantly increased barcode expression in Fig. 1G, with significance ($q < 0.05$) calculated from the Fisher's exact test with the Bonferroni correction. There were no significant gene ontology terms for guides that decreased barcode expression in the DNA to RNA comparison.

bioRxiv preprint doi: https://doi.org/10.1101/2024.06.21.600132; this version posted June 22, 2024. The copyright holder for this preprint (which was not certified by peer review) is the author/funder, who has granted bioRxiv a license to display the preprint in perpetuity. It is made available under aCC-BY 4.0 International license.

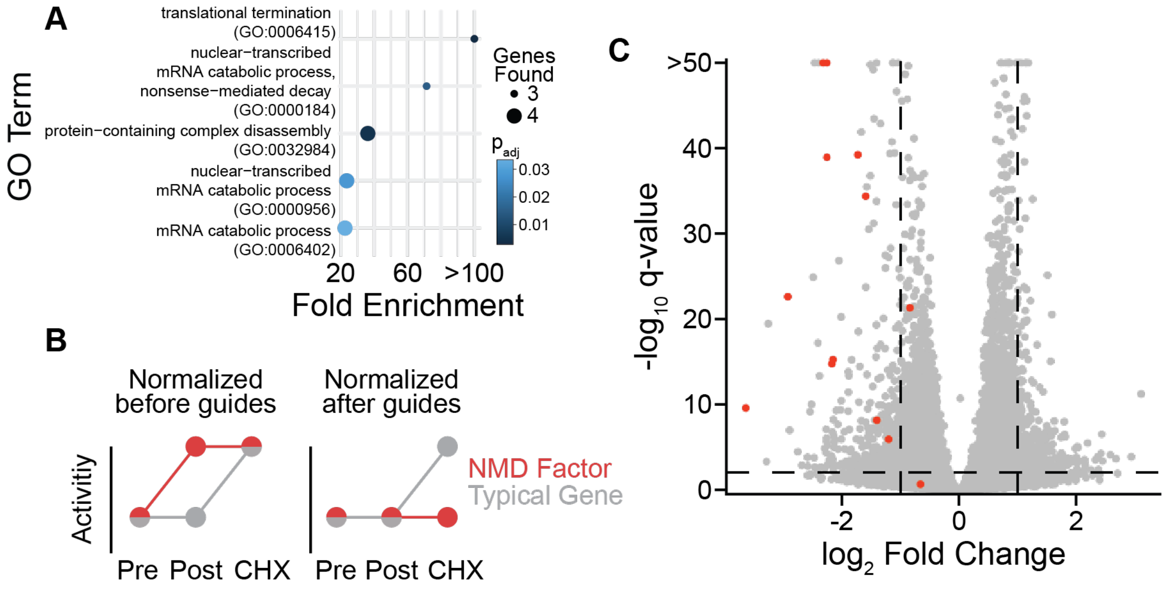

Figure S3: CiBER-seq profiles the requirement of activate translation for NMD activity. (A) Gene ontology enrichment analysis of guides that stabilize the PTC containing reporter compared to all guides from Fig. 4E. Significance ($q < 0.05$) was assessed with Fisher's exact test with the Bonferroni correction. No significant gene ontology terms were found for guides that appear to further destabilize the reporter. **(B)** Schematic of expected guide activities based on the comparisons in linear models. NMD factors should have lower activity than a typical gene in the cycloheximide treatment, because the reporter harboring the PTC is not further stabilized by drug treatment. **(C)** Analysis of genome-wide CiBER-seq screen for NMD factors after cycloheximide treatment, normalized to post-guide induction. Guides targeting NMD factors, as determined in Fig. 4E, are labelled in red. Dashed lines represent q-values < 0.01 and > 1 $\log_2$-fold change.
